# Isolation, characterization, therapeutic and prophylactic applications of a lytic bacteriophage to combat multi-drug resistance Shigella flexneri: an animal study model

**DOI:** 10.3205/dgkh000543

**Published:** 2025-04-30

**Authors:** Parisa Abbasi Fashami, Abazar Pournajaf, Nour Amirmozafari, Masoume Hallajzadeh, Vahid Pirhajati Mahabadi, Reza Saghiri, Soraya Khafri, Rezvan Golmoradi Zadeh, Sousan Akrami, Sajjad Asgharzadeh, Mehdi Rajabnia

**Affiliations:** 1 Infectious Diseases and Tropical Medicine Research Center, Babol University of Medical Sciences, Babol, Iran; 2 Department of Microbiology, School of Medicine, Tehran University of Medical Sciences, Tehran, Iran; 3 Neuroscience Research Center, Iran University of Medical Sciences, Tehran, Iran; 4 Department of Biochemistry, Pasteur Institute of Iran, Tehran, Iran; 5 Department of Social Medicine, Babol University of Medical Sciences, Babol, Iran

**Keywords:** S. flexneri, lytic bacteriophage, dysentery, therapeutic option

## Abstract

**Background:**

*Shigella (S.) flexneri* is one of the most important causes of disease in children with diarrhea in Iran. Today, bacteriophages are an attractive option to resolve the drug resistance problem among pathogenic agents. Accordingly, the present study aimed to isolate a lytic bacteriophage of *S. flexneri* and investigate its impact on reducing excretion of *Shigella* in mouse models suffering from bacillary dysentery.

**Method:**

*S. flexneri* ATCC12022 was used. Identification of the phage isolated from hospital wastewater was performed using Transmission Electron Microscopy (TEM). Stability tests were performed to determine the sensitivity of isolated phages to various factors such as temperature, pH and bile salts. A male Syrian mouse model (C57), with mice 6 weeks of age weighing 22–25 g, was used to ensure safety and efficacy of the bacteriophage in reducing *Shigella* in stool. Treatment with the phage was performed (I) 1 h before, (II) 1 h after, (III) 5 h after, and (IV) 1 h before +1 h after bacterial challenge.

**Results:**

TEM indicated that the bacteriophage used in this study belongs to the Myoviridae family. Administration of one dose of bacteriophage before the infection can accelerate improvement post-transfection, and administration of bacteriophage post-infection has a therapeutic influence.

**Conclusion:**

*In vivo* and *in vitro* results indicate that our bacteriophage causes complete lysis of *S. flexneri*. Thus, this phage could be a therapeutic option for treating bacillary dysentery resulting from multidrug-resistant *S. flexneri*.

## Zusammenfassung

**Hintergrund:**

*Shigella (S.) flexneri* ist eine der wichtigsten Krankheitsursachen bei Kindern mit Dysenterie im Iran. Bakteriophagen sind eine aussichtsreiche Option, um das Problem der Arzneimittelresistenz bei Krankheitserregern zu lösen. Dementsprechend zielt die vorliegende Studie darauf ab, lytische Bakteriophagen von *S. flexneri* zu isolieren und ihre Auswirkungen auf die Verringerung der Ausscheidung von *Shigella* in Mausmodellen mit *S. flexneri* Diarrhoe zu untersuchen.

**Methode:**

Es wurde *S. flexneri* ATCC 12022 verwendet. Die Identifizierung der aus Krankenhausabwässern isolierten Phagen erfolgte mit einem TEM-Elektronenmikroskop. Es wurden Stabilitätstests durchgeführt, um die Empfindlichkeit der isolierten Phagen gegenüber verschiedenen Faktoren wie Temperatur, pH-Wert und Gallensalzen zu bestimmen. Um die Sicherheit und Wirksamkeit der Bakteriophagen bei der Reduzierung von Shigellen im Stuhl zu gewährleisten, wurden männliche syrische Mäuse (C57) im Alter von 6 Wochen und einem Gewicht von 22–25 g verwendet. Die Behandlung mit Phagen erfolgte: (I) 1 h vor, (II) 1 h nach, (III) 5 h nach und (IV) 1 h vor +1 h nach der bakteriellen Infektion.

**Ergebnisse:**

Die in der Studie verwendeten Bakteriophagen gehören zur Familie der Myoviridae. Die Verabreichung einer Dosis des Bakteriophagen vor der Infektion kann eine Verbesserung des Verlaufs nach der Transfektion bewirken. Die Verabreichung von Bakteriophagen nach der Infektion hat einen therapeutischen Einfluss.

**Schlussfolgerung:**

Die Untersuchung des Bakteriophagen unter *In-vivo*- und *In-vitro*-Bedingungen zeigt, dass er eine vollständige Lyse von *S. flexneri* bewirkt. Dementsprechend kann der Phage eine potenzielle therapeutische Option für die Behandlung der durch multiresistente *S. flexneri* verursachten Diarrhoe sein.

## Introduction

Diarrhea resulting from bacteria, viruses, and parasites is a public health concern. Among diarrhea-causing pathogens, *Shigella* spp. play a significant role in developing bloody and inflammatory diarrhea [1]. For instance, *Shigella flexneri* is one of the most important causes of this disease among children suffering from diarrhea in Iran [2]. *Shigella* pathogenesis is based on the bacterial ability to attack and proliferate in the colon’s epithelium, causing severe inflammation and epithelial degeneration. More than 164 million cases are reported annually, with 1.1 million mortalities, in response to shigellosis in developing countries; most patients are children under five years of age [3]. Most *Shigella* infections respond to antibiotic treatment, but increasing consumption of antibiotics has led to the emergence and spread of antibiotic-resistant strains, which has led to the development of multi-drug resistance, making it a serious concern of the World Health Organization [4]. Thus, finding novel alternatives for infection control is essential. Bacteriophages (or phages) specifically attack bacterial cells, causing their death; however, they do not affect human or animal cells [5]. Phage therapy involves the targeted usage of bacteriophages for treating infections resulting from bacteria, which in case of interacting with the target bacteria, can infect and kill them [5]. Unlike antibiotics, the approach of phages is limited and does not disrupt the normal equilibrium of the intestinal microflora [6]. That is, they only cause minor damage to the beneficial bacteria of the body, such as intestinal and lactic-acid bacteria [7]. Meanwhile, phages function in a constrained way, and after killing the harmful bacteria, they enter the inactive phase of their lifecycle and show virtually no activity against non-host bacteria. They are not allergens and have only minor side effects [8], [9]. Considering the therapeutic properties of phages, the present study aims to isolate specific lytic bacteriophages of *S. flexneri* and explore their impact on reducing *Shigella* excretion in a mouse model suffering from bacillary dysentery.

## Materials and methods

This cross-sectional study was performed within a 10-month period from May 2020 to February 2021 and was approved by the research ethics committee of Babol University of Medical Sciences (code IR.MUBABOL.REC.1399.101).

### Source of S. flexneri and isolation and purification of the phage

*S. flexneri* ATCC 12022 was obtained from the Microbiology Department of IUMS (Iran University of Medical Sciences), Tehran, Iran. To isolate the specific lytic phage of *S. flexneri*, the typical isolation protocol was used with some modifications [6]. A wastewater sample was collected from the first basin of the Motahari hospital wastewater treatment plant and utilized as the source of the phage. All samples were collected after centrifuging at 2,500×g for 20 min, and then filtered through a syringe filter (45.0 µm). Then, 10 ml of the bacteria in the logarithmic phase (4-h fresh culture) was added to 10 ml of treated wastewater as well as 10 ml Brain Heart Infusion (BHI) culture medium with double concentration (2×). The mixture was incubated overnight in a shaker incubator at 37°C and 90 rpm. Then, the suspension was centrifuged and the supernatant was filtered (45.0 µm). The presence of the specific lytic phage in the suspension was explored using the double-layer agar (DLA) method. Emergent transparent plaques were considered to demonstrate the presence of the lytic phage of *S. flexneri*. A plaque was chosen, and the purification method was performed three times consecutively. The phage was typically replicated according to the protocol of Sambrook and Russell [10].

To determine the titer of phages, pure and filtered lysates of phages were used to prepare serial dilutions, after which DLA was used. The obtained plaques were counted, whereby the titer of phages was calculated according to the following equation:

pfu/ml=number of plaques/dilution factor × phage.

To eliminate the residuals of bacteria and to obtain a more concentrated phage sample, the phage suspension (43.3×10^11^ pfu/ml) was centrifuged (Beckman optima XL-90 Ultracentrifuge) at 4,000×g for 120 min; thereafter, the residue was dissolved in SM buffer. Then, the phage suspension was placed in a copper grid with carbon coating (Ted Pella Inc, USA), after which negative staining was performed with phosphotungstic acid (PTA) 2%. Finally, the sample was examined using transmission electron microscopy (TEM; Zeiss LEO 906 (Germany) [11].

### Stability studies

To determine the sensitivity of the isolated phages, stability tests were performed with some modifications. To determine the stability at different temperatures, lysates (diluted at a 1:100 ratio) were exposed to 40–80°C for 60 min, and to –20°C as well as –80°C for 48 hours. To evaluate their stability, the different titers were deplaqued using the DLA method and the phage changes were compared against the stock sample [12].

To evaluate the stability at different pHs, different amounts of 1 n NaOH and 1 n HCl were added to the BHI medium to obtain the desired pH (1–3, 5–7, 9–11). Next, 100 µL of the phage suspension diluted at a 1:100 ratio was added to 900 µL of each BHI medium with a different pH. The samples were incubated for 1 hour or 16 hours in the incubator at 37°C. Thereafter, to evaluate their stability by DLA method, plaques were obtained from their different titers, and phage changes were compared with the original titer [11], [13].

To evaluate the stability of the phage in bile salt, BHI medium containing 1% and 2% concentrations of oxgall powder (bovine bile, Sigma-Aldrich, Germany) was used. 100 µL of the phage suspension diluted at a 1:100 ratio was added to 9.9 mL of medium containing 1% or 2% bile, and then incubated at 37°C for 1 or 3 hours. After the incubation, the phage suspension was prepared as a serial dilution and phage survival was examined using the double-layer agar technique [14].

### Determination of phage adsorption extent

The bacteria were cultured in two flasks and incubated at 37°C until reaching OD_600nm_=0.2–0.5 nm. Phage diluted at a 1:100 ratio was added to one of the flasks with the same amount of bacterial culture. The second flask with no phages was used as the control. Both flasks were placed inside a shaker-equipped incubator at 37°C and 130 rpm. Using a spectrophotometer (Beckman Coulter), OD_600nm_ was measured for each flask once every 10 min for 2 hours [11], [15].

### One-step growth curve

The host bacteria were incubated in the BHI culture medium until reaching the logarithmic phase (OD_600__nm_=0.4–0.5 nm). 6 mL of 4-hour bacterial culture plus 6 mL phage suspension diluted at a 1:100 ratio was incubated for 15 min inside a shaker incubator at 37°C. Thereafter, the suspension was centrifuged for 10 min at 10,000×g to precipitate the bacteria. The resulting sediment was washed three times with normal saline and transferred to a falcon tube. All falcon tube were placed inside the shaker incubator at 37°C and 130 rpm. 1,000 µL was withdrawn from each falcon tube and centrifuged every 10 min for 2 hours, then passed across a 45.0-µm filter. Ultimately, after preparing the serial dilutions, each sample was used to investigate the plaque formation using the DLA method.

### Effect of the isolated phage on S. flexneri in vivo

To prepare the bacterial strain required for the formation of bacillary dysentery disease in the mice, *S. flexneri* spp. ATCC 12022 was incubated in BHI medium until reaching OD_600nm_=0.8–1 nm in a shaker incubator at 37°C at 150 rpm. To prepare the bacteriophage for treatment, phage lysates were centrifuged at 4,000 rpm for 30 min and then filtered (22.0 µm).

As the model host, male Syrian mice C57 (6 weeks old, weight 22 g±3.2 g), acquired from the IUMS experimental study center, were used. The mice were fed freely and routinely, and had access to fresh water around the clock. The animal studies were conducted based on the guidelines of the Ethics Committee for Animal Experimentsat IUMS. 

To evaluate the effectiveness of phage therapy, the mice were examined in five groups:

1. Group I: The prophylaxis group received phage 1 h before bacterial force-feeding;
2. group II: treatment group in which the phage was administered after transfection with *Shigella*;
3. group III: treatment group which received phage 5 hours after bacterial force-feeding;
4. group IV: treatment group which received phage 1 hour before and 1 hour after bacterial force-feeding;
5. group V: control group (received bacteria only) [16].

200 µL bacteria and an equal amount (200 µL) of phage were force-fed to the mice. In group IV, 100 µL phage was force-fed before and 100 µL after bacterial force-feeding. Separate sterile needles were used for bacterial and phage force-feeding in order to prevent confounding results. 

### Colony count

Fresh stool was collected from all groups on days 1 and 3. The stools were investigated in terms of appearance and weight, and then colony count was done for them according to the study by Mai et al. [17] . Biochemical tests were used to confirm that the strains were *Shigella*. 

### Statistical analysis

SPSS software version 25 (SPSS, Chicago, IL, USA) was used to analyze the data. Kruskal-Wallis and adjusted Bonferroni ad-hoc tests were applied to determine correlation between variables. p≤0.05 was considered statistically significant.

## Results

Plaque formation indicated the presence of lytic phage against *S. flexneri*. Fully transparent plaques formed, having diameters of 0.3–0.8 mm. One of the plaques was chosen, enriched, and used for subsequent tests. According to TEM results, the isolated phage had an icosahedral head with 80 nm diameter, attached directly to the 131-nm-long non-contracting tail. Based on the morphological features, this phage was classified as a member of the *Myoviridae* family, order *Caudovirales*. 

Also, according to the equation mentioned in the methods section, the phage titer was determined to be 43.3×10^11^. 

### Effect of temperature on phage survival

**Figure 1:**
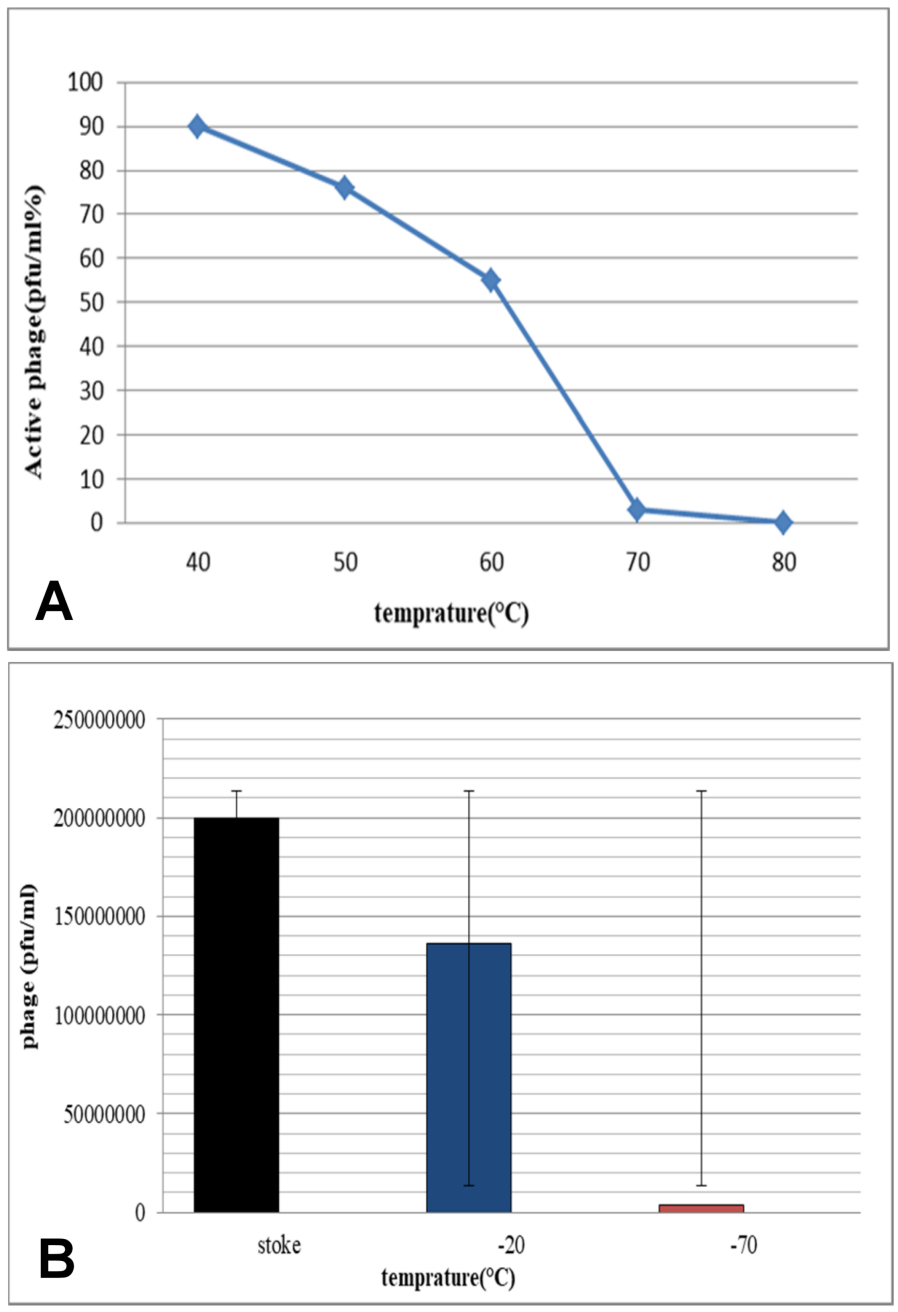
A: Measurement of the bacteriophage resistance at 40–80°C; B: Comparison of phages at –20 and –70°C against the stoke sample

To analyze the stability of phages at high temperatures, freephages were exposed to 40–80°C. The results showed that they are resistant to heat up to 60°C, but their number approaches zero at 70°C. Most of these phages appear to be inactive at 80°C (Figure 1A (Fig. 1)). One method of long-term storage involves freezing. Examination of the stability of phages at –20 and –70°C showed that keeping phages at –20°C does not significantly alter their biology, but at –70°C, the number of phages is reduced. Phages begin to proliferate again by removing them from –20 and –70°C and adding fresh culture of the host bacterium (*S. flexneri*) plus incubation at 37°C for 24 hours (Figure 1B (Fig. 1)).

### Effect of pH on phage survival

**Figure 2:**
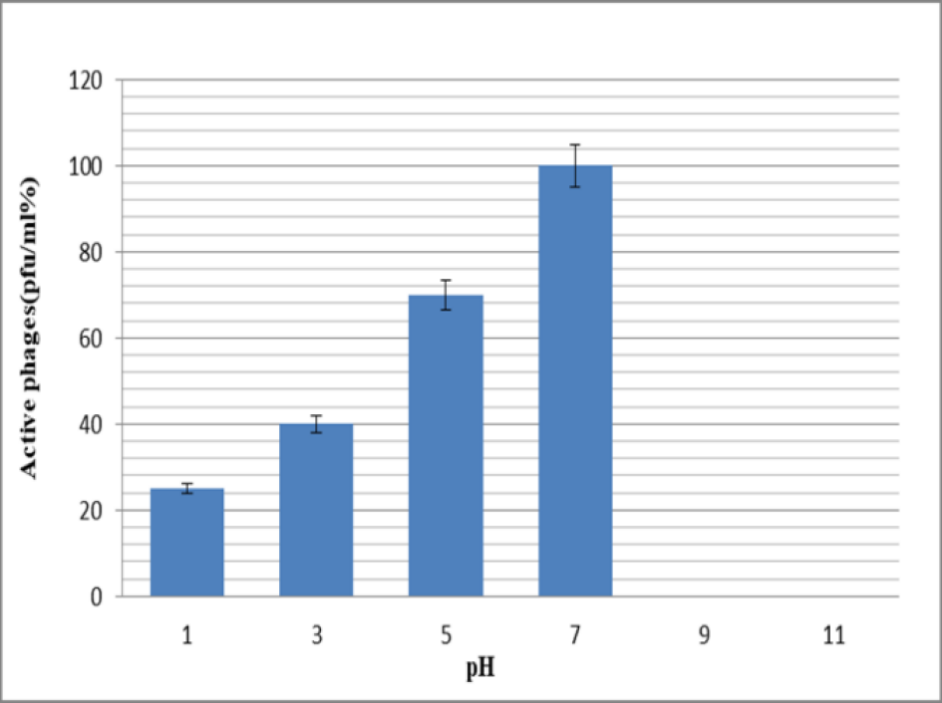
Survival rate of the bacteriophages at various pHs within 1 hour

The pH resistance test showed that during incubation of phages for 16 hours, plaque is formed only at pH=7 (2×10^7^ pfu/ml). However, in 1 h incubation, the phage had good stability at acidic pHs, while the number of phages was undetectable at pHs 9 and 11. The results showed that high pH may present a barrier to the phage (Figure 2 (Fig. 2)).

### Stability of the phages in bile salts

**Figure 3:**
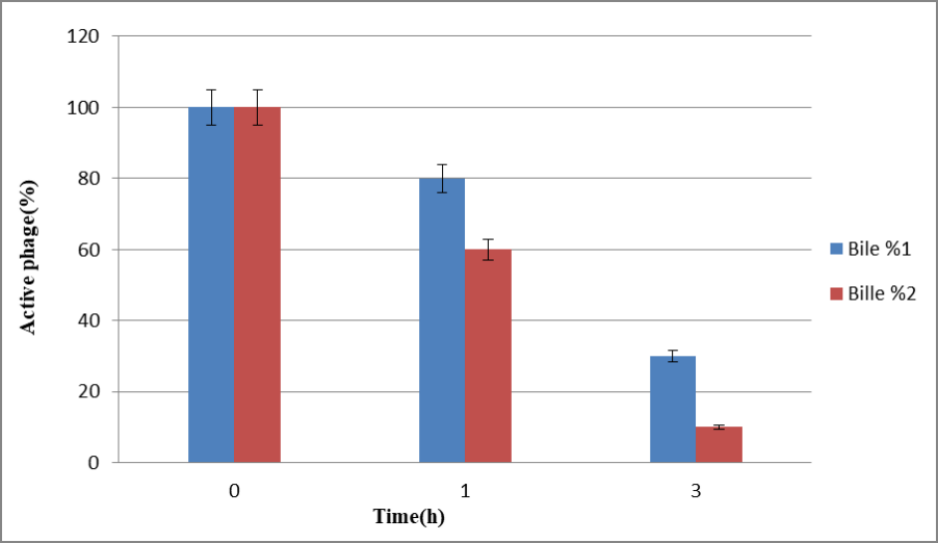
Comparison of bacteriophage resistance in 1% and 2% bile within 1 and 3 h

Bacteriophages showed better stability in 1% bile within 1 hour and 3 hours compared to 2% bile (Figure 3 (Fig. 3)).

### Bacterial reduction assay for the phage

**Figure 4:**
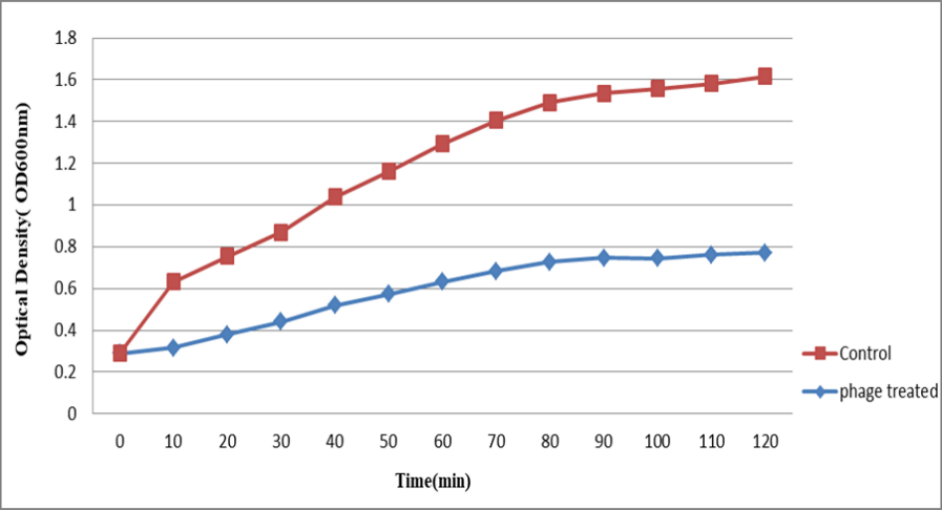
Extent of absorption of bacteriophages over time

To determine the bacterial reduction assay of bacteriophages, OD was measured for both falcons (one of them as control) once every 10 min for 2 hours (to observe bacterial lysis). The results of this investigation showed that with an increase in the control OD (bacterial increase), the phages also revealed an ascending trend (Figure 4 (Fig. 4)).

### The results of one-step growth curve

**Figure 5:**
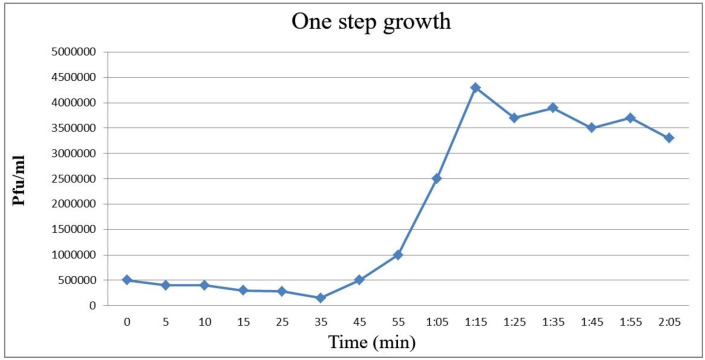
Single-stage growth of the *S. flexneri* phage

This test was done to determine the phage’s incubation period and burst size. Based on the results, the incubation period was 35 min, and phage burst size was 377 pfu/cell per infected cell. The burst size was calculated based on the mean ratio of the phage performance of the bacterial cells to the average released particles of the infected phage (Figure 5 (Fig. 5)) [13].

### Effect of the phage on S. flexneri in vivo

**Figure 6:**
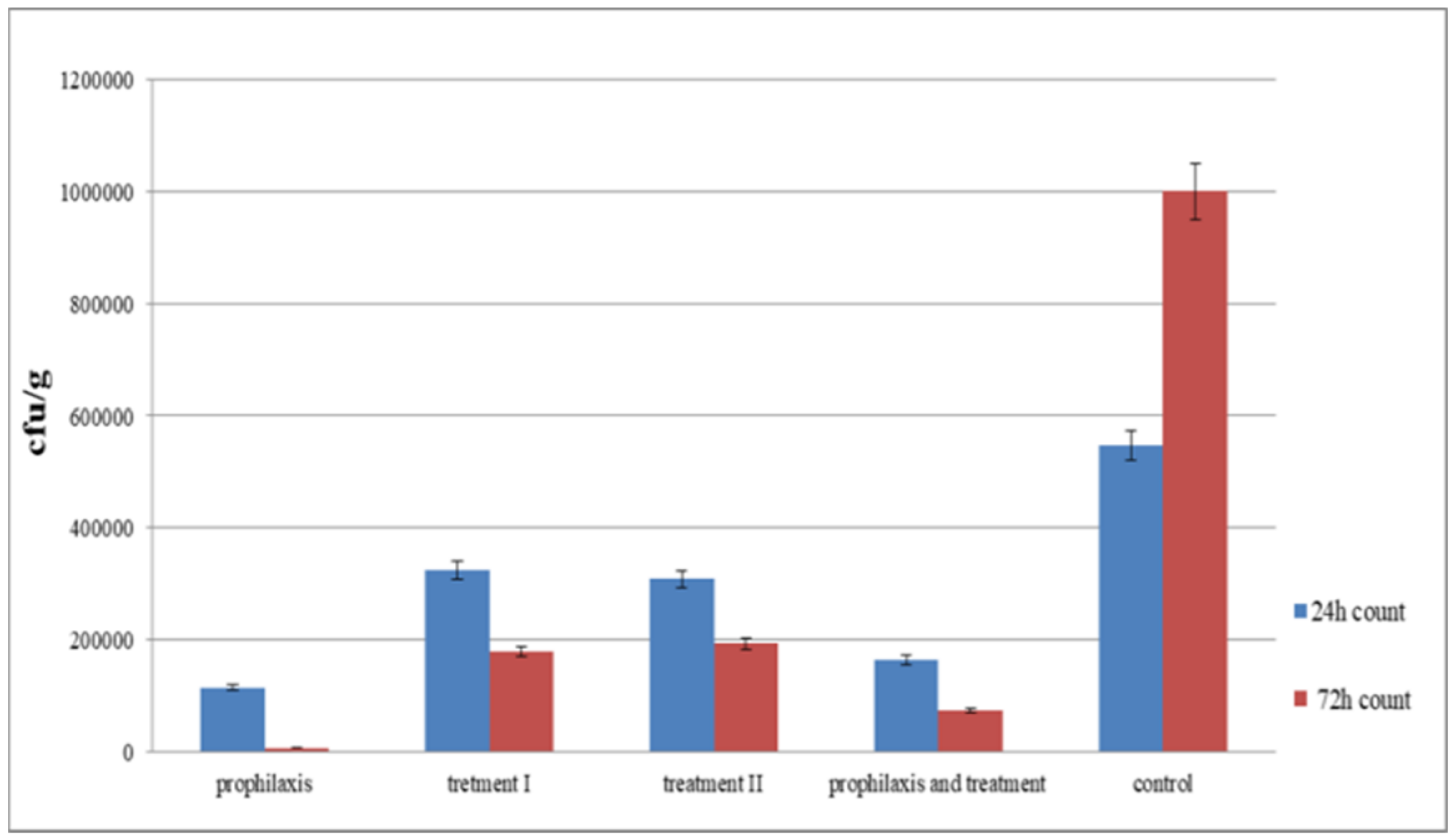
Assessment of the colony forming number of *S. flexneri* in five different groups after 24 hours

The groups of mice that received phage showed greater improvement within 24 hours compared to control group. Among phage-receiving groups, group I followed by group IV had the most significant improvement (Figure 6 (Fig. 6)). After 72 hours, fresh stool samples were collected again from the mice groups. Before homogenizing into normal saline, the samples were visually examined and weighed. Serial dilution was prepared from this suspension for colony counting. It was dispersed uniformly on a MacConkey agar plate using a Pasteur pipette, and incubated overnight at 37°C.

**Figure 7:**
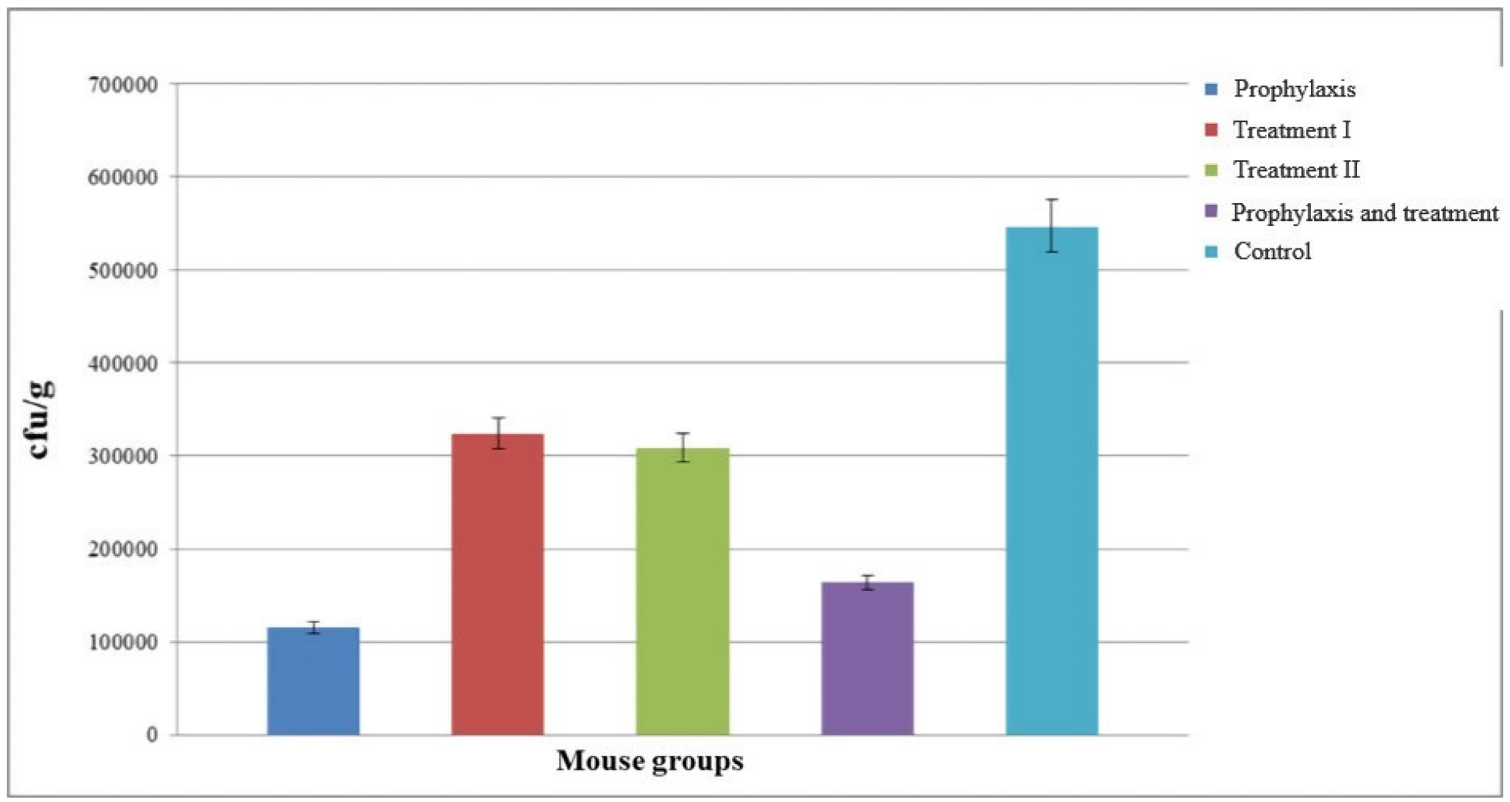
Assessment of colony forming number of *S. flexneri* in five different groups after 72 hours

Colonies were counted the next day and recorded as colony forming units (cfu)/g. Compared to the control group, a decrease in the number of cfu’s was observed in all groups of mice. The greatest reduction was observed in group I, followed by group IV (Figure 7 (Fig. 7)). 

**Figure 8:**
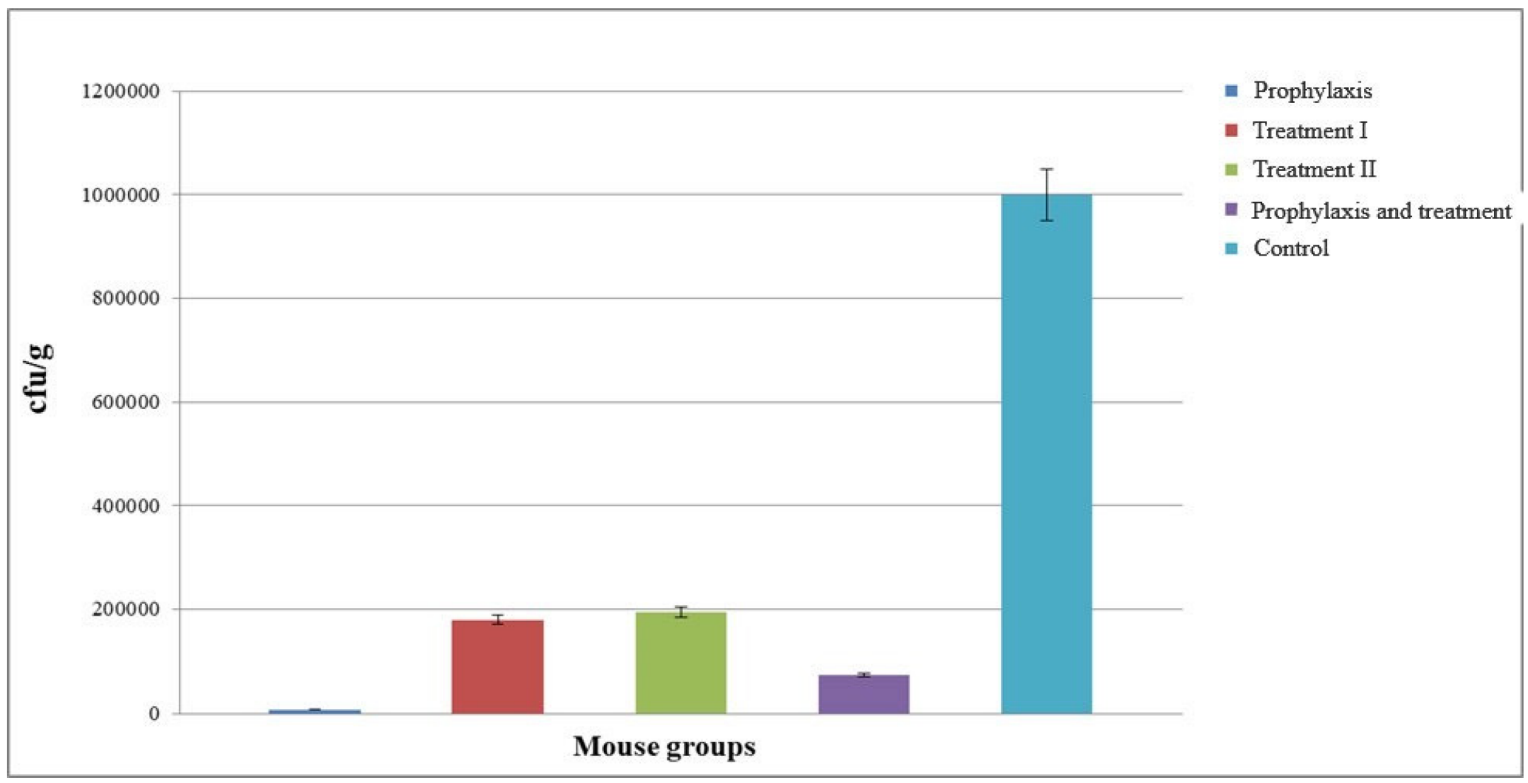
Comparison of the number of colony forming unit of *S. flexneri* in different mouse groups on days 1 and 3

The number of cfu’s of *S. flexneri* across different mouse groups on days 1 and 3 indicates that the number of cfu’s decreased on the third day, showing which had considerable improvement over time compared to the control group. Group I, followed by group IV, showed the greatest improvement (Figure 8 (Fig. 8)).

## Discussion

Diarrhea resulting from the *Shigella* bacterium is generally called shigellosis [18]. Clinical and epidemiological studies suggest that *Shigella* is one of the major causes of diarrhea, especially in children under five, with the dominant *Shigella* species in developing countries being *S. flexneri* [19]. The disease is usually transferred from person to person or through food and water [20]. In industrial countries, milder cases are more common, while more severe cases and often fatal dysentery occur in developing countries. Considering the high transmissivity of the disease due to low infective dose (200 cfu’s), and since humans are considered the only reservoir of *Shigella*, a control treatment-based program is required alongside observing hygienic principles [21]. The emergence of antibiotic-resistant strains has led to the failure of effective treatments against shigellosis as well as other bacterial infections, potentially increasing health-care costs as well as mortality [16]. Despite efforts of more than a century, a certified vaccine for *Shigella* is not yet available [18]. Therefore, finding an alternative strategy for controlling infection is essential. Indeed, phages come to the forefront because of the emergence of antibiotic-resistant bacteria [22], and shigellosis is one of the first human diseases for which treatment outcome has improved using phage therapy [23]. Mikeladze et al. [24] reported 50% reduction in mortality of bloody diarrhea patients through phage therapy. Anpilov et al. [25] also reported a tenfold decrease in incidence of bloody diarrhea in patients treated with phages.

The studied phage was isolated from the wastewater of Shahid Motahari burn accident hospital (the first basin of the treatment plant). According to some studies, wastewater per se and hospital wastewater in particular generally contains a large diversity of microorganisms due to contaminated excreted materials [26]. The isolated phage was highly lytic and produced transparent plaques ranging from 0.3 to 0.8 mm in diameter. The morphological characteristics indicated that the isolated phage belonged to the *Myoviridae* family, *Caudovirales* order. 

Several studies have documented that phages differ from each other in terms of pH and heat stability. Investigating the stability of the studied phage across a wide range of temperatures and pH is important. This study showed that the phage could tolerate temperatures up to 65°C, while at 70°C, their numbers dropped to almost zero. It seems that most of these phages are inactive at 80°C, which is due to the protein coating of the phage. Furthermore, the stability of the present phage at –20°C offers a perfect condition under which phages may be kept easily. Numerous studies have reported that most phages can survive within a wide pH range of 5 to 9, maintaining the native viral structure and stability [27]. However, the *Myoviridae* phage isolated in this study had good stability for 1 hour at low pH values. This indicates that the mentioned phage can tolerate the acidic pH of the stomach, and oral administration of the phage has no significant effect on its lytic activity. Therefore, assessment of phage survival through the digestive system is essential. Accordingly, stability measurements in 1% and 3% bile were performed for the mentioned phage for periods of 1 and 3 hours. It was found that the studied phage has relatively good stability in the bile salts, although when exposed to the simulated intestinal fluid, slight negative effects were observed on the survival of the free phages.

The longer the incubation period of the phage, the larger the burst size will be. The burst size of the phage is the average number of phages produced after infection in a bacterial cell [28]. In 2015, Zhang et al. [29] isolated a high-risk phage from *Lacticaseibacillus (L.)* casei ATCC 393 and reported an incubation period of about 75 min, which followed by a relatively short burst period of 45 min. The burst size was approximately 16/pfu per each infected cell at 30°C. In 2017, Sunthornthummas et al. [30] reported a 55-min incubation period for *L. paracasei* phage ΦT25 at 37°C, which decreased with a 55 min incremental period and average burst size of around 38 phage particles in each infected cell. For the studied phage, a single-stage growth curve was drawn, showing a biphasic curve that identified the incubation period and burst size of the phage. According to the single-stage growth curve, the phase incubation period was 35 min, and its burst size was 377 pfu/cell phage particles in each infected cell. The large burst size of the phage in this study indicates its suitability for use in treatment. After 40 min, a new generation of phages starts to generate and eventually a large number of new phages are produced. These features suggest the therapeutic potential of the studied phages. These phages seem to produce a large number of new generation phages within 40 min, and fulfill the need for high concentration of phages and high doses for treatment.

The interaction of phages and host cells plays a significant role in practical applications. The effectiveness of phage activity is associated with the host cells, which causes an increased probability of binding of phages to the host cells. If the phage reaches the site of bacterial infection, it will be effective in eliminating the infection. When the bacterial infection is systemic and the bacteria are scattered throughout the animal’s body, it seems likely that ensuring a sufficient number of phages would enhance the therapeutic effect, as it increases the probability of targeted binding of the phage to the bacteria [31]. The first stage in the phage infection process is binding and absorption, which determines the specificity [32]. In our study, in which OD was measured for the phage and control samples for two hours, this phage considerably reduced the bacterial number under *in vitro* conditions (Figure 5 (Fig. 5)). Concurrent with an increase in the number of host bacteria, the phage also begins to grow. Thus, we used this phage for* in vivo* experiments. 

To observe the effectiveness of the isolated phages *in vivo*, a mouse model was employed for prophylaxis and treatment of bacillary dysentery. Our observations indicated that phage therapy was highly effective for prophylaxis. Meanwhile, the comparison of the five groups using the Kruskal-Wallis test (p<0.05) indicated that all five groups were significant together, while for pair-wise comparison of the groups, the adjusted Bonferroni ad-hoc test was utilized, which showed pair-wise significance. Groups I and IV, representing the prophylaxis and prophylaxis plus treatment, respectively, showed the greatest statistically significant differences (p<0.001) compared to other groups. 

Phage therapy is developing as a promising treatment of infections resulting from multi-drug resistant bacteria. There are numerous reports about phages that have been isolated and therapeutically implemented against antibiotic-resistant *Shigella* pathogens [13], [22]. The results of prophylaxis and treatment with the phage studied here showed that administration of the phage either before or after infection yields quite similar effects. Furthermore, *in vivo* methods demonstrated that the free phages can be recovered from the stool in very small amounts following oral administration to the mice. These results confirm studies showing that phages survive passage through the gastrointestinal tract and can be excreted from animal feces in a dose-dependent manner. Some researchers have proposed that phages enter the bloodstream of the laboratory animals within 2 to 4 hours (following one oral dose), and are found in internal organs (liver, spleen, kidneys, etc.) within 10 hours. Also, the data related to the persistence of the administered phages indicate that the phages can remain in the body for a relatively long time, i.e., several days [33]. Although phages can circulate well in the bloodstream and in different organs, some researchers have shown that phages may be neutralized by antibodies, thereby inhibiting the targeted lysis of bacteria [34]. According to Sulakvelidze et al. [35], the production of neutralizing antibodies should not be a major obstacle during the primary treatment of acute infections, as the phage kinetic of action is far faster than the production of the host’s neutralizing antibodies. In addition, if the phage neutralizing antibodies still exist during implementation of the second course of treatment or if a rapid immune response occurs before phages exert their action, by re-administering the phage or elevating its concentration, this problem can be overcome [35]. 

Overall, our results present definite proof of effective treatment of bacillary dysentery in mouse models using phages, which is in line with several other reports. It clearly indicates that phages can function as a therapeutic agent against bacterial infections. Previous studies have shown that compared to antibiotic therapy, phage therapy presents more effective results, and it seems to be a safe agent to replace antibiotics [36]. Although phage therapy is a promising substitute for antibiotics against special and treatment-resistant infections, precise assessment of its therapeutic potential is an absolute prerequisite before clinical application [37].

## Notes

### Acknowledgements

We would like to thank Babol University of Medical Sciences for funding this study. 

### Authors’ ORCIDs

- Mehdi Rajabnia: 0000-0002-5343-0878
- Sousan Akrami: 0000-0001-6643-140X

### Ethical approval

The study was approved by the research ethics committee of Babol University of Medical Sciences; Babol, Iran ( code IR.MUBABOL.REC.1399.101).

### Funding

The study was financially supported by Babol University of Medical Sciences (grant number: 744132621).

### Competing interests

The authors declare that they have no competing interests.
